# Surgery for mycotic aneurysm

**DOI:** 10.1186/1749-8090-8-S1-O29

**Published:** 2013-09-11

**Authors:** Z Mitrev, T Anguseva, E Idoski, E Stoicski

**Affiliations:** 1 Special Hospital for Surgery Fillip II, Skopje, Macedonia

## Backgound

Infected aneurysm (or mycotic aneurysm) is defined as an infectious disease of the wall of an artery with formation of a blind, saccular out-pouching that is contiguous with the arterial lumen. Symptoms are frequently absent or non-specific during the early stages. Once clinically presented, infected aneurysms are often at an advanced stage of development and associated with complications such as rupture. Nontreatment or delayed treatment of infected aneurysms has a poor outcome, with high morbidity and mortality rate of fulminant sepsis or hemorrhage. In clinically suspected cases, computed tomography is state –of-the-art technique used for diagnosis. Imaging features of infected aneurysms include a lobulated vascular mass, an indistinct irregular arterial wall, perianeurysmal edema, and a perianeurysmal soft-tissue mass. Urgent surgery performed for solving aortic rupture, carries high morbidity and mortality rates.

## Methods and results

Our experience with a 24 years old women suffering from mycotic aneurysm is related with three successive operations. Successful treatment was accomplished by aneurismal resection and in situ prosthetic reconstruction.

## Conclusion

The best chance of survival in patients with this difficult condition depends on early computed tomography evaluation and prompt surgical intervention under appropriate and intensive antibiotic therapy.

